# Transcriptome-based stemness indices analysis reveals platinum-based chemo-theraputic response indicators in advanced-stage serous ovarian cancer

**DOI:** 10.1080/21655979.2021.1939514

**Published:** 2021-07-16

**Authors:** Xinwei Sun, Qingyu Liu, Jie Huang, Ge Diao, Zhiqing Liang

**Affiliations:** a Department of Gynecology and Obstetrics, Southwest Hospital, Army Medical University, Chongqing, China; b Orthopedic Department, The 964th Hospital of Chinese People’s Liberation Army Joint Logistics Support Force, Changchun, China; c Department of Obstetrics and Gynecology, Daping Hospital, Army Medical University, Chongqing, China

**Keywords:** SOC, mRNAsi, WGCNA, TCGA, stemness, platinum response

## Abstract

Serous ovarian cancer (SOC) is a main histological subtype of ovarian cancer, in which cancer stem cells (CSC) are responsible for its chemoresistance. However, the underlying modulation mechanisms of chemoresistance led by cancer stemness are still undefined. We aimed to investigate potential drug-response indicators among stemness-associated biomarkers in advanced SOC samples. The mRNA expression-based stemness index (mRNAsi) of The Cancer Genome Atlas (TCGA) was evaluated and corrected by tumor purity. Weighted gene co-expression network analysis (WGCNA) was utilized to explore the gene modules and key genes involved in stemness characteristics. We found that mRNAsi and corrected mRNAsi scores were both greater in tumors of Grade 3 and 4 than that of Grade 1 and 2. Forty-two key genes were obtained from the most significant mRNAsi-related gene module. Functional annotation revealed that these key genes were mainly involved in the mitotic division. Thirteen potential platinum-response indicators were selected from the genes enriched to platinum-response associated pathways. Among them, we identified 11 genes with prognostic value of progression-free survival (PFS) in advanced SOC patients treated with platinum and 7 prognostic genes in patients treated with a combination of platinum and taxol. The expressions of the 13 key genes were also validated between platinum-resistant and -sensitive SOC samples of advanced stages in two Gene Expression Omnibus (GEO) datasets. The results revealed that CDC20 was a potential platinum-sensitivity indicator in advanced SOC. These findings may provide a new insight for chemotherapies in advanced SOC patients clinically.

## Graphical abstract

**Figure:**
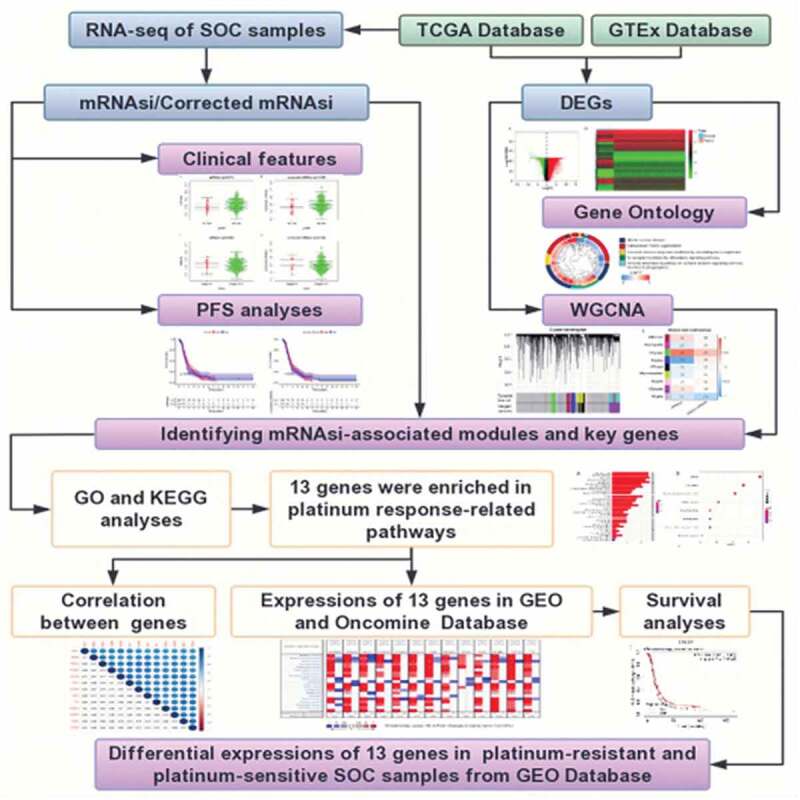

## Introduction

Ovarian cancer (OC) is the leading lethal malignancy occurred in female reproductive organs. Serous ovarian cancer (SOC) is a distinct histological subtype of OC and is often diagnosed at advanced stages [1]. Poor drug response often leads to a disappointing prognosis of SOC patients. In recent years, the hypothesis of cancer stem cells (CSCs) has been widely accepted. CSCs possess potential features of self-renewal and uncontrolled growth [2]. The subpopulation of cells has persistently maintained the competence of self-perpetuation and simultaneously given rise to differentiated types of progeny tumor cells through asymmetrical division [2]. The stemness of CSCs is an important cause of tumor chemoresistance as well as a potential target of anticancer strategies [3,4]. Investigating stemness-associated genes in advanced SOC might be feasible to explore drug response indicators [5].

In the last decade, high-throughput technology has achieved a considerable amount of data storage in public databases and provided high-quality data for deeper data mining. Therefore, machine learning has been successfully applied to the medical fields, particularly in oncological research [6]. To summarize the features of stem cells, Malta et al. [7] used a one-class logistic regression (OCLR) machine learning algorithm to extract transcriptomic feature sets from normal tissue-derived pluripotent stem cells and their differentiated progeny, which have different degrees of stemness. They identified stem cell signatures and quantified stemness by using transcriptome data. Ultimately, a stemness index, mRNAsi was proposed in their study. The researchers had further analyzed cancer stemness in 33 tumor types in TCGA to verify mRNAsi scores. Based on the study, we obtained the mRNAsi of each SOC sample in TCGA to further utilize in our study.

In the present study, the clinical significance of mRNAsi and identified stemness-associated genes in advanced SOC samples from TCGA were explored by using mRNAsi and WGCNA. Function annotation and pathway enrichment analysis were conducted to recognize platinum response-associated pathways and genes. Potential platinum response indicators would be selected from the identified genes by survival analyses and validation on multiple databases, which provided a possible hypothesis for screening advanced SOC patients who are more likely to have a positive response to platinum-based chemotherapy. In summary, we aimed to investigate the cancer stemness-associated genes by bioinformatic methods and selected potential platinum-response indicators among them by prognostic value and expression level. This study may provide new clues for drug response prediction to guide platinum administration in advanced SOC patients.

## Materials and methods

### Data acquisition and pre-processing

The RNA sequencing (RNA-seq) expression data used in this study were downloaded from the UCSC Xena project (https://xena.ucsc.edu/) based on February 2020. The datasets included tumor tissue samples from TCGA (N = 379 for SOC) and normal ovarian tissue (N = 88) from GTEx. Both cohorts have been previously recomputed to minimize differences from distinct sources based on a standard pipeline. The corresponding clinical information was downloaded from the TCGA-OV dataset (https://portal.gdc.cancer.gov/). The mRNAsi indices of 273 SOC samples in TCGA were obtained from a previous study [4]. Perl language (https://www.perl.org/) was used to convert gene IDs to gene symbols. The RNA-seq data of the included normal and tumor samples were combined into a matrix file by R language.

### Clinical feature correlation analysis of mRNAsi and corrected mRNAsi

The tumor purity score of the SOC samples in TCGA was obtained from a previous study [8]. Corrected mRNAsi was calculated by the mRNAsi score/tumor purity score. A total of 262 tumor samples with available information of mRNAsi and 254 samples with corrected mRNAsi score were included in the correlation analyses with histopathological grades, and clinical stages. Wilcox test was performed using a beeswarm package to determine the significant difference between the two groups. Removed the samples with incomplete information of survival time, PFS analyses were conducted on 240 SOC patients of stage III–IV. The prognostic significance of mRNAsi and corrected mRNAsi was explored by survival and surviminer packages in R.

### Differentially expressed genes (DEGs) analysis

The DEGs were screened with RNA-seq data in TCGA-OV and GTEs cohort. The limma package was applied to perform the differential expression analysis and the Wilcox test was used to determine the significant difference in the processing [9]. |Log2 Fold change (FC)| >1 and False Discovery Rate (FDR) <0.05 were the criteria to screen the DEGs between normal and tumor samples. Heatmap and volcano plot was drawn using the heatmap package. The Gene Ontology (GO) terms were visualized by the GO plot package.

### Identification of key genes by WGCNA

The co-expression network of DEGs according to mRNAsi was constructed by WGCNA package in R [10]. The R packages ‘matrixStats’, ‘foreach’, ‘Hmisc’, ‘doParallel’, ‘fastcluster’, ‘dynamicTreeCut’ and ‘survival’ were also applied in this process. Following the removal of normal and SOC samples of stage I–II, a total of 352 SOC samples remained for subsequent analysis. Samples were clustered with the average method according to the gene expression level. The cut-height was set as 100 and the minimum size of gene groups was set as 10 to exclude the outlier. In this procedure, 15 outlier samples were removed and 337 samples were included in the subsequent analyses. The pre-processed data was intersected with the mRNAsi data and analyzed.

The optimal power-value was selected to construct a scale-free network according to the Pearson correlation coefficient among genes. The power-value was then determined as 8 by calculating the correlated genes between the scale-free R^2^ and mean connectivity. A GeneTree was constructed with the power-value and the dynamic module was identified with a minimum gene size of 50. Adjacent modules were merged with the criteria MEDiss Thres <0.25. The module-trait correlations with mRNAsi and EREG-mRNAsi were plotted.

After selecting modules of our interest, we calculated the gene significance (GS, a correlation between gene expression levels and sample traits) and module membership (MM, a correlation between genes in a certain module and gene expression profiles for each gene). To obtain more possible enriched pathways, we defined cor. gene MM>0.80 and cor.gene GS>0.4 instead of 0.50 [11] as the thresholds to obtain more potential key genes.

### Function annotation and pathway enrichment analysis of key genes

The Gene Ontology (GO) and Kyoto Encyclopedia of Genes and Genomes (KEGG) enrichment analysis were performed and visualized by clusterProfiler R package, enrichplot, and ggplot2 [12]. The candidate key genes were eventually selected according to the enriched pathways.

The PPI network was constructed using the STRING platform (Version 11.0, https://string-db.org/) [13]. The minimum required interaction score was set as medium confidence (0.4). We calculated the number of adjacent nodes to show the connectivity of each protein in the PPI network. The Pearson’s correlation coefficient between the paired key genes was computed according to the gene expression levels and visualized by the corrplot package [11]. The results with a correlation coefficient >0.4 were considered a strong correlation between the paired genes.

### Data validation

The significant differential expression of the selected key genes was showed by heatmap and ggpubr package. We further selected two datasets GSE18520 [14] and GSE69428 [15], from the GEO database (https://www.ncbi.nlm.nih.gov/geo/), to validate the differential expression level of the selected key genes. The GSE18520 dataset included 10 normal ovarian surface epithelium samples and 53 advanced, high-grade SOC samples. The GSE69428 dataset included 10 high grade serous ovarian cancer (HGSOC) samples and 10 paired normal oviducts samples. DEG analyses were conducted with a limma package. Oncomine (https://www.oncomine.org/) was utilized to investigate the mRNA expression levels of the key genes at a pan-cancer level.

### Prognostic and chemotherapeutic response predict the value of key genes

Survival analyses of key genes were conducted with the Kaplan Miere Plotter (https://kmplot.com/). The prognostic value of the key genes on OS was examined in SOC patients of all stages in the database. The impact of the selected key genes on the OS and PFS was examined on SOC patients of stage III–IV receiving platinum or the combination of platinum and taxol. A *p* value <0.05 was considered statistically significant. In addition, GSE131978 [16] and GSE51373 [17] datasets were selected to validate whether the key genes were associated with platinum sensitivity in advanced SOC patients. In GSE131978, a total of 7 platinum-based chemotherapy-resistant SOC samples of stage III–IV and 4 platinum-based chemotherapy-sensitive samples were selected for analysis. In GSE51373, 10 platinum-resistant and 13 platinum-sensitive samples of stage III–IV were also utilized in the validation.

## Results

### The prognostic roles and clinical characteristics of mRNAsi/corrected mRNAsi in SOC

To obtained the platinum-based chemotherapeutic response indicators in advanced-stage SOC, we first analyzed the correlation between mRNAsi/corrected mRNAsi scores and clinical characteristics in SOS samples. Subsequently, PFS analyses on mRNAsi/corrected mRNAsi were performed to reveal the prognostic value of advanced SOC patients. Next, DEGs between normal ovarian samples and SOC samples were screened. WGCNA was applied to distinguish mRNAsi-associated modules and genes. Then, The platinum response-associated pathways and key genes were selected by GO and KEGG analyses. Furthermore, a series of expression validations and prognostic value analyses of key genes were conducted using multiple databases. Finally, the differential expressions of key genes between platinum-resistant and -sensitive SOC samples were identified using two GEO datasets.

**Figure 1.**
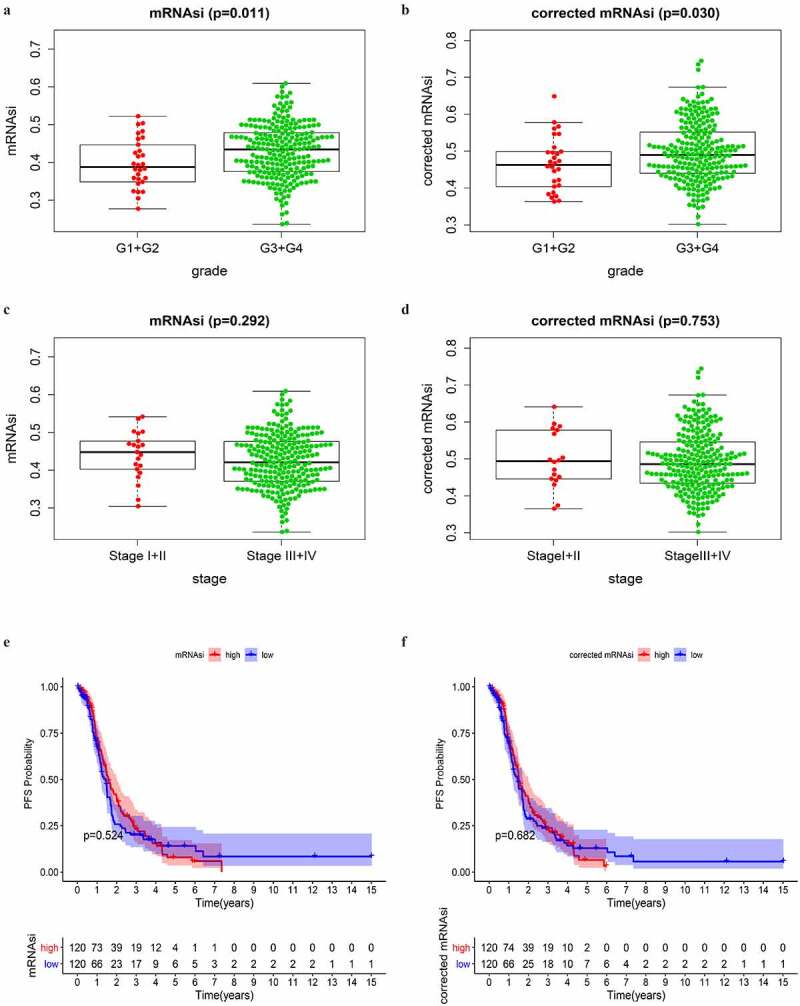
Correlations between mRNAsi/corrected mRNAsi and clinical characteristics in SOC of stage III–IV. (a, b) The clinical correlation of mRNAsi and corrected mRNAsi with histopathological grades; (c, d) The clinical correlation of mRNAsi and corrected mRNAsi with stages; (e, f) PFS analyses of mRNAsi and corrected-mRNAsi among SOC patients of stage III–IV; p < 0.05 indicates statistical significance

The mRNAsi index was reported to be derived from normal cells and cells with different degrees of stemness via calculating on the TCGA transcriptomic data [7]. It could be considered a quantitative marker of CSCs stemness. Tumor tissues were composed of different kinds of cells, including tumor cells and other types of cells, such as stromal and immune cells. Tumor purity was considered an interference factor affecting the evaluation of the mRNAsi score. Here we obtained the tumor purity score of OC from a previous study, which had calculated the score of multiple cancers in TCGA [8]. The mRNAsi was corrected as previously reported (mRNAsi/tumor purity) [11]. The correlation analyses between mRNAsi/corrected mRNAsi and clinical features were performed on all SOC patients in TCGA. Because of the extremely small sample size of Grade 1 and Grade 4, as well as stage I and stage IV in the TCGA database, we divided the SOC samples into two groups according to histopathological grades (G1+ G2 and G3+ G4) and clinical stages (stage I–II and stage III–IV), respectively. As shown in Figure 1(a,b), SOC samples of higher grades had greater mRNAsi and corrected mRNAsi score than those of lower grades with statistical significance. However, mRNAsi and corrected mRNAsi scores were not significantly associated with stages (Figure 1(c,d)). These results indicated that cells in SOC samples of higher grades had greater stemness than those of lower grades.

As reported, CSCs were the main cause of tumor chemoresistance. PFS was commonly used as a surrogate of chemotherapy response in OC [17]. Thus, we conducted PFS analyses on mRNAsi and corrected mRNAsi to reveal whether they had prognostic value among SOC patients of stage III–IV. SOC cases of stage III–IV with complete available information of PFS time were divided into low and high score groups based on the mRNAsi or corrected mRNAsi scores. We observed that there was no significant difference in PFS between low and high score groups (Figure 1(e,f)). More unexpectedly, advanced SOC patients with greater mRNAsi scores, which indicated stronger stemness of the tumor cells, had an even higher PFS rate within approximately 4 years showed in the survival curve. Similar results were observed in the analysis on corrected mRNAsi. These findings were different from our understanding that greater stemness of tumor cells would indicate decreased PFS [18]. The above results indicated that the mRNAsi score in SOC samples had a close correlation with the histopathological grades. The corrected procedure didn’t obviously impact the results of clinical correlation. Therefore, mRNAsi, instead of corrected mRNAsi, was used in the subsequent analyses.

### DEGs between normal ovarian and SOC tissues

**Figure 2.**
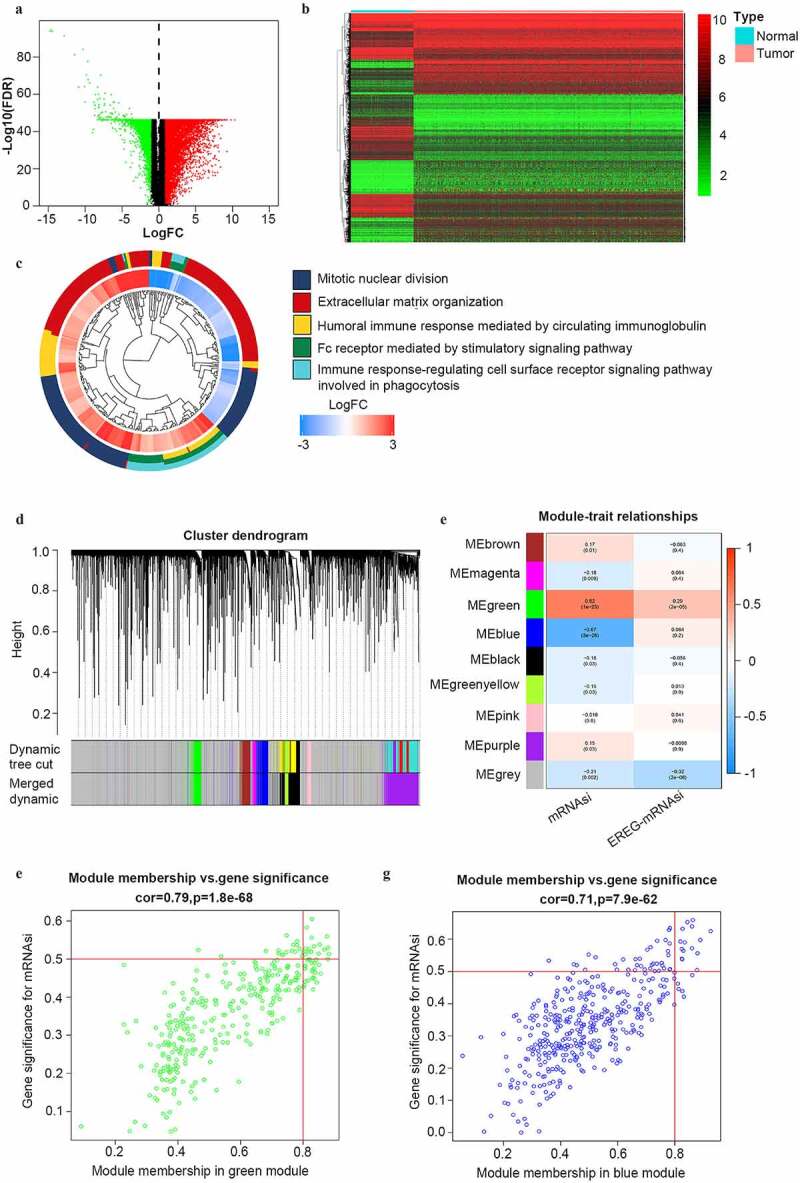
Identification of DEGs and stemless-associated gene modules in SOC of stage III–IV. (a, b) Volcano plot and heatmap of DEGs.Red represents upregulated genes; green represents downregulated genes and black represents genes without significant upregulation or downregulation; (c) The GO categories of the DEGs screened between normal ovarian tissue from GTEx and all SOC samples from TCGA; (d) Identification of weighted gene co-expression modules in SOC of stage III–IV. Each piece of the leaves on the cluster dendrogram matched a certain gene. Genes with similar expression patterns compose a branch; (e) Correlations between gene modules and mRNAsi or EREG-mRNAsi in SOC of stage III–IV. The upper row in each cell indicates the correlation coefficient quantifying the correlation between a certain gene module and the corresponding mRNAsi or EREG-mRNAsi score. The lower row in each cell indicates the p value; (f, g) The scatter plots of the top modules positively correlated and negatively correlated to mRNAsi: the green module and the blue module. Each colored circle represents a gene and the circles located in the upper right square indicate the key genes in the corresponding module. p < 0.05 indicates statistical significance

To reveal the potential stemness-associated key genes according to mRNAsi, we screened DEGs between normal ovarian samples and SOC samples of all stages (N = 379). Thus, we identified 7255 DEGs, including 3790 upregulated ones and 3465 downregulated ones (Figure 2(a,b)). Among the GO categories (Figure 2(c)), ‘mitotic nuclear division’ was the most enriched biological process (BP) of the DEGs.

### Identification of mRNAsi-related modules and key genes by WGCNA

To identify mRNAsi-associated modules and key genes, WGCNA was used to construct a co-expression network to cluster the samples and DEGs into biological modules. In the study, outlier samples were first eliminated from the analysis (Figure S1A) and the remained samples of stage III–IV (N = 337) were clustered according to mRNAsi and EREG-mRNAsi score (Figure S1B). β = 8 (scale-free R^2^ = 0.950) was selected as the soft threshold to ensure the scale-free network (Figure S1C). Thus, we obtained 9 biological gene modules (Figure 2(d)).

To explore the relationship between gene modules and the mRNAsi scores, we used module significance (MS) to quantify the correlation between the overall gene expression level of the corresponding module and mRNAsi. The upper row of each module was the R^2^ value, representing the degree of correlation between gene expression and mRNAsi or EREG-mRNAsi in the corresponding module (Figure 2(e)). According to the R^2^ value, the green module was most positively associated with mRNAsi (with a correlation coefficient close to 0.80, p value = 1.8e-68) and the blue module was most negatively associated with mRNAsi (with a correlation coefficient of 0.71, p = 7.9e-62) (Figure 2(f,g)). To study the key genes positively correlated to stemness characteristics of advanced-stage SOC, we chose the green module for further study. To avoid missing some crucial enriched pathways caused by a lack of included genes, the criteria were defined as cor. MM > 0.80 and cor. GS > 0.40 (not 0.50 as reported [11]) to acquire full information of enriched pathways. Finally, we obtained 42 key genes including AURKA, AURKB, BIRC5, BUB1, CCNA2, CCNB2, CDC20, CDC6, CDCA5, CDK1, CENPA, CKAP2L, DEPDC1, DLGAP5, ECT2, ERCC6L, EXO1, FAM83D, HJURP, KIF15, KIF18B, KIF23, KIF2C, KIF4A, MCM10, MELK, MYBL2, NCAPG, NCAPH, NDC80, NEK2, NUF2, NUSAP1, ORC1, PLK1, RACGAP1, RRM2, SGO1, TOP2A, TPX2, TTK, and UBE2C.

### Function annotation and pathway enrichment of the selected key genes

**Figure 3.**
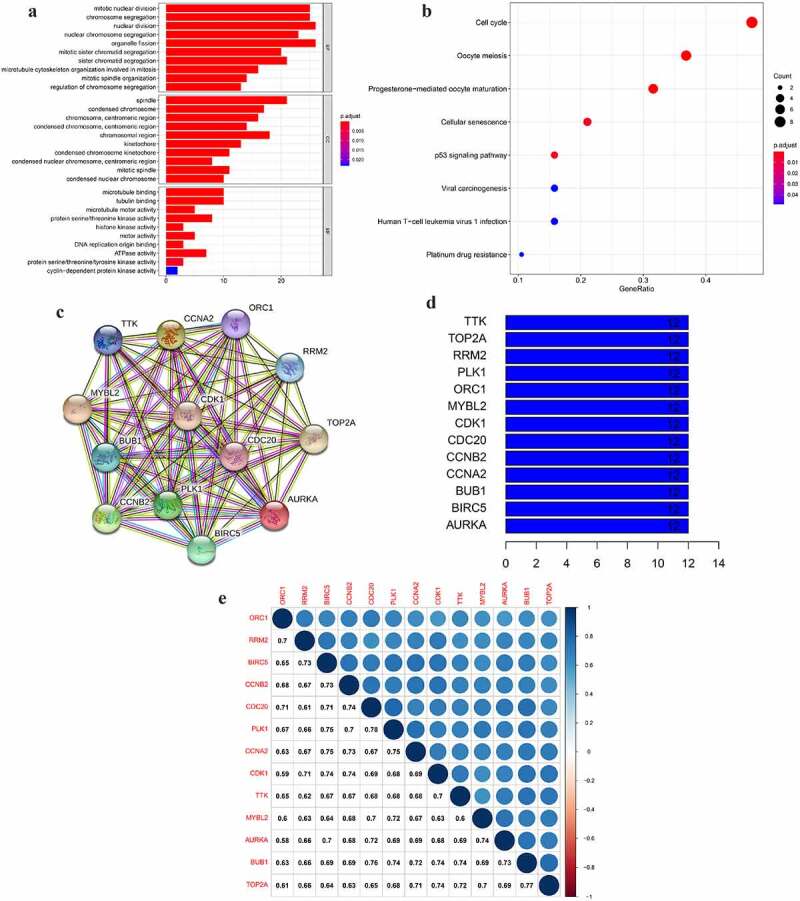
The GO and KEGG analyses and PPI network of the selected key genes. (a, b) The top GO categories and KEGG results of 42 selected key genes; (c) The protein–protein interaction network of 13 selected key genes according to the KEGG pathway enrichment results; (d) The number of edges of each key gene in the PPI network; (e) Correlation coefficient between paired key genes at the transcriptional level

To elucidate the biological process and signaling pathways the selected key genes involved in GO and KEGG analyses were performed. The results revealed that the top 5 BP of the green module was mainly associated with cell mitosis and proliferation (Figure 3(a)). These key genes were mainly involved in the ‘cell cycle’ pathway (Figure 3(b)). Interestingly, the selected key genes were also enriched to the platinum response-associated pathways ‘cellular senescence’ [19,20], ‘p53 signaling pathway’ [21,22], and ‘platinum drug resistance’ (Figure 3(b)). Cell cycle arrest was also the main molecular mechanism of the platinum anticancer effect [23,24]. To explore the potential platinum-based therapeutic response indicator, we obtained a total of 13 key genes enriched to the four aforementioned pathways to be further analyzed in advanced SOC.

### Correlation between key genes at mRNA and protein level

To explore the mutual correlation of the key genes and their protein products, we used the Pearson correlation method and STRING online tool to perform the analysis. As shown in Figure 3(c), each node represented a protein in the network. The PPI network showed a wide and strong relationship among the encoded proteins of the selected key genes. We calculated the edge (Figure 3(d)) and found every node in the network was connected to the rest 12 proteins, indicating a mutual correlation with all of the rest proteins. At the mRNA level, the relationship between PLK1 and CDC20 had the highest correlation coefficient of 0.78. BUB1 and TOP2A, as well as BUB1 and CDC20, had a lower correlation coefficient of 0.77 and 0.76. AURKA and ORC1 had the lowest correlation coefficient of 0.58 (Figure 3(e)). These results demonstrated that the selected key genes composed a strong and dense interaction network.

### Expressional validation of key genes in multiple datasets

**Figure 4.**
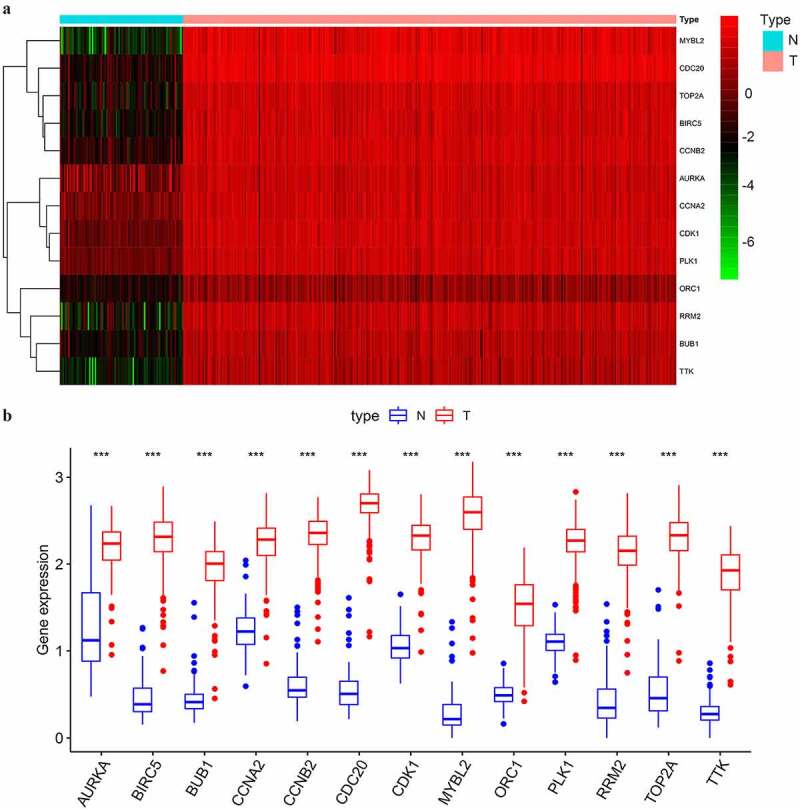
The differential expression of the selected key genes in the green module. (a) The heatmap showed the expression level of selected key genes among 88 normal ovarian samples and 352 SOC samples of stage III–IV. Samples were divided into two groups, normal (N), and tumor (T). Red indicated a high expression level and green indicates a low expression level; (b) The average expression level of the selected key genes visualized by boxplots. Blue indicated the normal group and red indicates the tumor group. ‘***’ indicates p < 0.001

**Figure 5.**
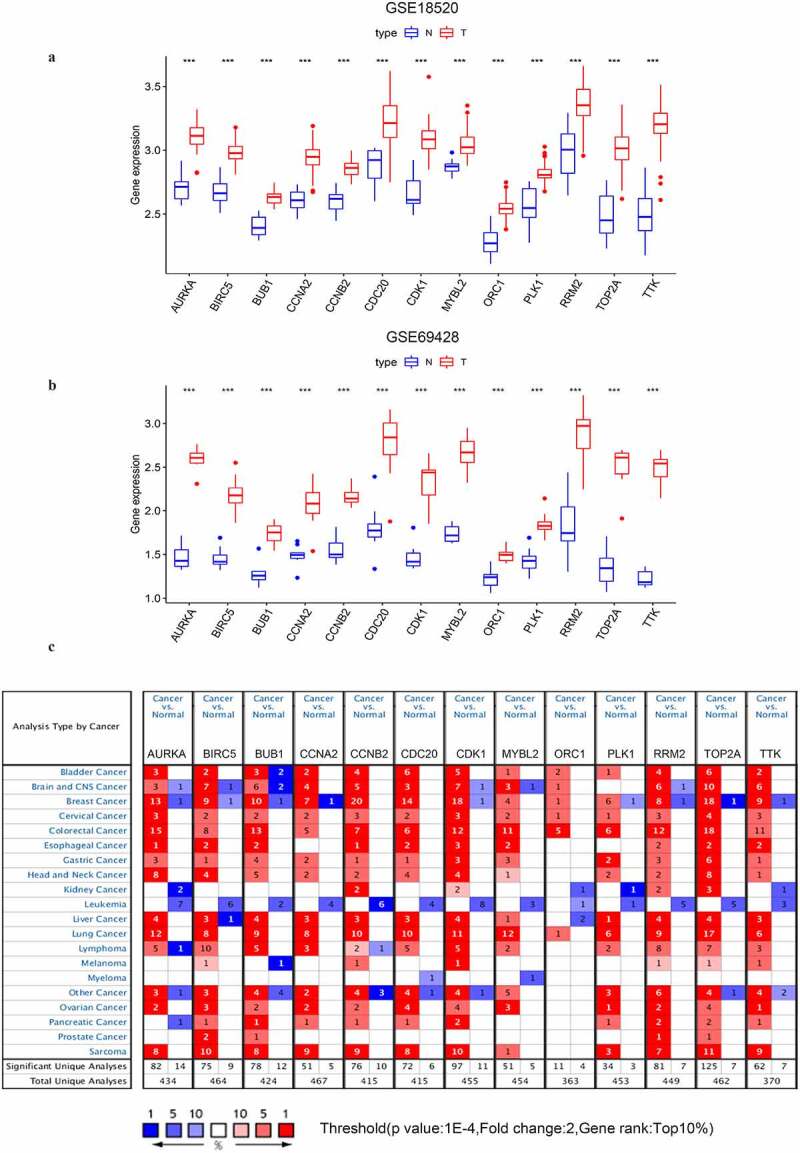
The expressional validation of selected key genes in different databases. (a, b) Expression level of the key genes in the GSE18520 and GSE69428 datasets of the GEO database. ‘**’ indicates p < 0.01. ‘***’ indicates p < 0.001; (c) The mRNA expression of key genes in multiple cancer types in Oncomine database. The number in the cells represents the number of analyzed datasets in which the expression level of genes meets the thresholds shown below the graph. Red indicates a higher expression level of the certain gene in tumor tissues than the normal tissues. Blue indicates the opposite expression pattern. The color depth of each cell indicates the gene rank. The deeper the color depth, the higher the gene rank

As shown in Figure 4(a,b), the 13 selected key genes all had significantly higher expression levels in SOC samples of stage III–IV than in normal ovarian samples (p < 0.001). To verify the overexpression of the key genes in SOC samples, we selected two GEO datasets for validation. We first compared the expression of the key genes between normal ovarian samples and advanced stage, high-grade SOC samples in GSE18520, and confirmed that all the key genes were significantly upregulated (p < 0.001) in SOC tissue (Figure 5(a)). However, the origin of SOC was located in the fallopian tube rather than ovarian epithelium [15]. Therefore, we also evaluated the differential expression of the selected key genes between paired normal oviducts and advanced-stage SOC samples in GSE69428. All of the 13 key genes were significantly overexpressed in SOC tissue compared to normal oviducts (p < 0.001) (Figure 5(b)). These results further confirmed that the overexpression of the screened key genes in SOC tissue from the perspective of in-situ growth and tumorigenesis.

To further understand the expression levels of the key genes in multiple cancer types, we used Oncomine to perform the pan-cancer analyses. Except ORC1 was not in the top 10% gene rank of DEGs, the other 12 key genes were all ranked in the top 10% gene within at least one OC dataset (Figure 5(c)). The results strongly indicated that these key genes might be consistent oncogenes or even consistent stemness biomarkers widely overexpressed in multiple cancer types.

### The prognostic value and drug response indication of the key genes

**Figure 6.**
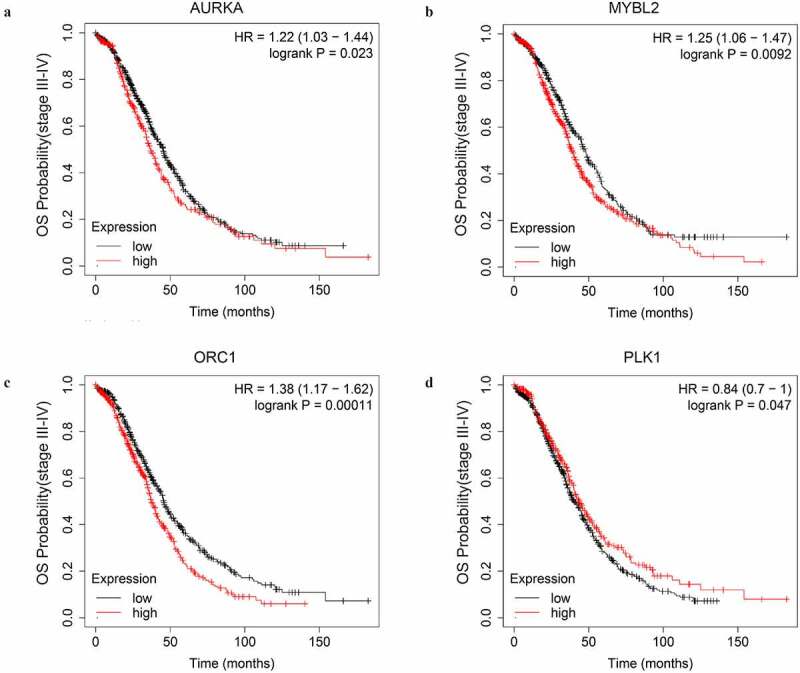
The OS curves of the key genes with significant prognostic value analyzed on 1023 SOC patients of stage III+IV by Kaplan-miere plotter

First, we explored the OS prognostic role of the key genes by Kaplan-Meire plotter. The results revealed that AURKA, MYBL2, ORC1, and PLK1 were associated with the OS of advanced-stage SOC patients with statistical significance. Higher expression level of AURKA, MYBL2, and ORC1 could predict shorter OS while the higher expression level of PLK1 could predict a longer OS (Figure 6, Table 1).

**Table 1.** The significant impact of the key genes on the overall survival time of the advanced-stage, SOC patients as well as patients treated with different strategy of chemotherapy

	Groups (patients)		Median OS (months)		
Gene expression	Low	High	Low	High	Difference
Stage III–IV, SOC AURKA MYBL2 ORC1 PLK1 Platinum-based AURKA CCNA2 MYBL2 ORC1 Platinum+taxol CCNA2 CDK1 ORC1 TOP2A TTK	678 425 533 677 610 306 390 485 352 157 364 236 425	345 598 490 346 326 630 546 451 220 415 208 336 147	45.13 48.00 45.73 40.00 45.77 40.54 48.27 46.13 45.63 50.00 45.47 48.37 45.63	36.73 38.57 37.03 43.93 38.77 44.13 39.77 37.93 40.00 41.60 38.57 41.00 38.47	8.40 9.43 8.70 –3.93 7.00 –3.59 8.50 8.20 5.63 8.40 6.90 7.37 7.16

**Figure 7.**
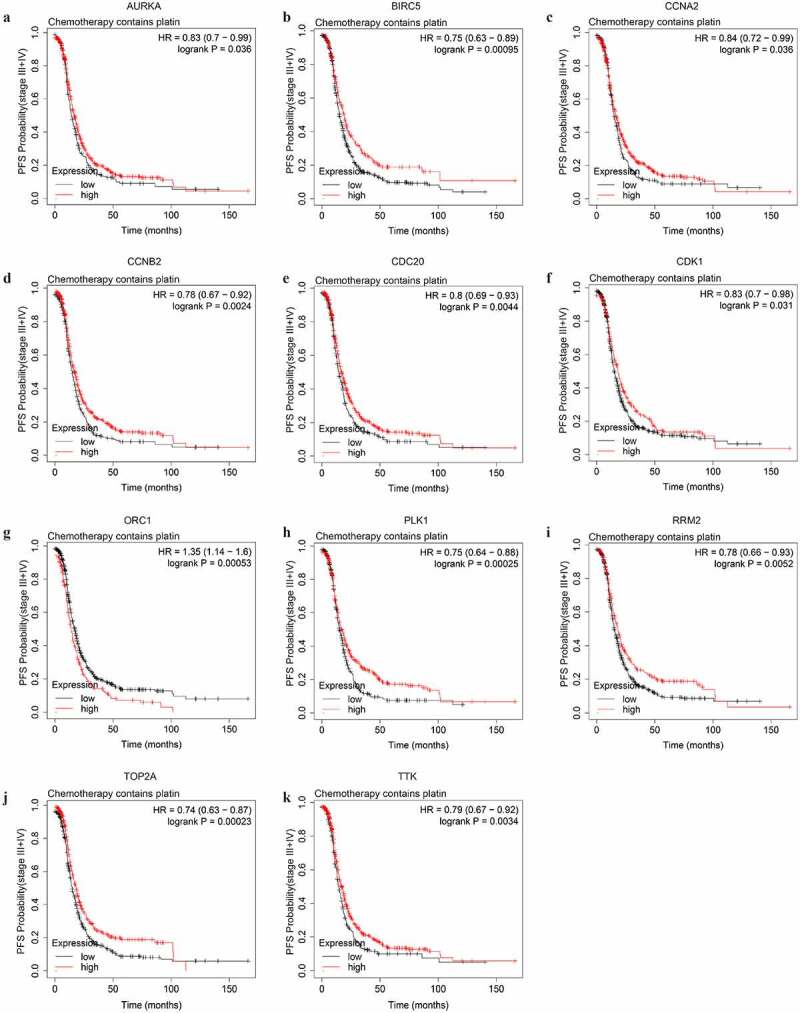
The PFS curves of the key genes with significant prognostic value analyzed on 907 SOC patients of stage III+IV treated with chemotherapy containing platin

To explore potential indicators of platinum-based chemotheraputic response among the stemness-associated key genes, we conducted OS and PFS analyses on the key genes among stage III–IV patients of SOC receiving platinum or the combination of platinum and taxol. Among the 13 key genes, the expression level of only 4 genes (AURKA, CCNA2, MYBL2, and ORC1) significantly affected the OS of platinum-treated advanced-stage SOC patients (Figure S2). The higher expression level of AURKA, MYBL2, and ORC1 could predict shorter OS while the higher expression level of CCNA2 could predict longer OS. The median OS difference of AURKA, MYBL2, and ORC1 between both the low and high expression groups was longer than 6 months, indicating the three genes were significant prognostic biomarkers of advanced-stage SOC. The expression level of 11 key genes (AURKA, BIRC5, CCNA2, CCNB2, CDC20, CDK1, ORC1, PLK1, RRM2, TOP2A, and TTK) were significantly associated with PFS (Figure 7). Among the prognostic genes of PFS, except for ORC1, the higher expression level of additional 10 genes could predict longer PFS (Table 2).

**Table 2.** The significant impact of the key genes on the PFS time of advanced-stage, SOC patients treated with different strategy of chemotherapy

	Groups (patients)			Median PFS (months)	
Gene expression	Low	High	Low	High	Difference
Platinum-based AURKA BIRC5 CCNA2 CCNB2 CDC20 CDK1 ORC1 PLK1 RRM2 TOP2A TTK Platinum+taxol BIRC5 CCNB2 CDC20 MYBL2 PLK1 TOP2A TTK	226 639 294 290 348 651 678 475 649 560 269 358 191 249 140 292 378 341	681 268 613 617 559 256 229 432 258 347 638 204 371 313 422 270 184 221	14.00 15.00 15.00 14.83 14.37 15.00 17.00 15.00 15.00 15.00 14.37 15.00 14.37 14.00 14.27 15.00 15.00 14.03	16.63 19.00 16.53 16.93 16.85 18.30 14.00 17.00 18.23 18.30 17.00 17.50 16.13 16.37 16.00 16.23 16.83 18.00	−2.63 –4.00 -1.53 –2.10 -2.48 –3.30 3.00 –2.00 -3.23 –3.30 -2.63 –2.50 -1.76 –2.37 -1.73 –1.23 -1.83 –3.97

**Figure 8.**
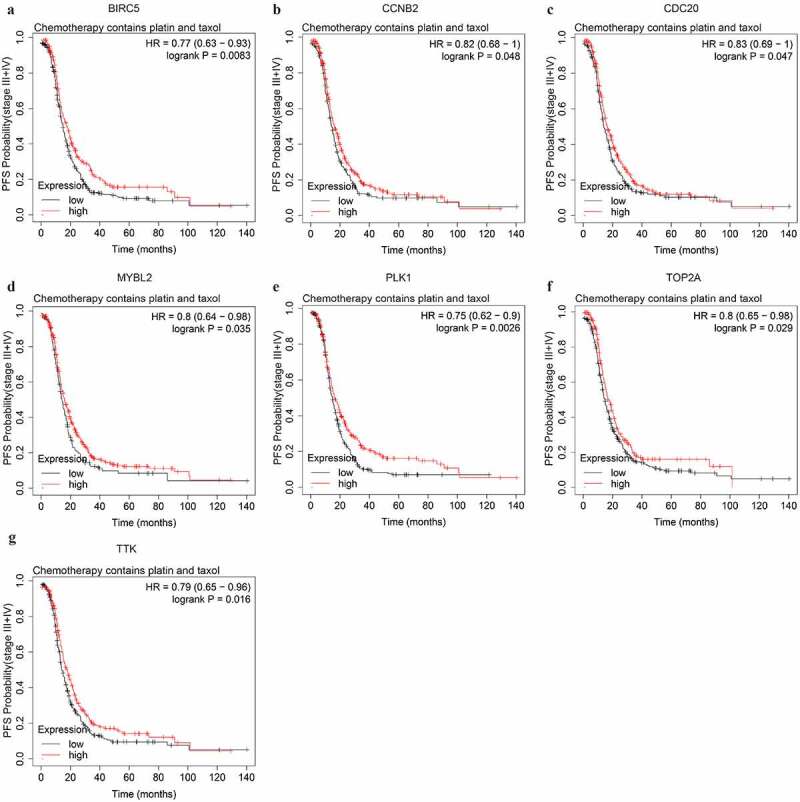
The PFS curves of the key genes with significant prognostic value analyzed on 562 SOC patients of stage III+IV treated with chemotherapy containing both platin and taxol

We further explored potential response indicators of chemotherapy containing both platinum and taxol by OS and PFS analyses. The expression level of CCNA2, CDK1, ORC1, TOP2A, and TTK were significantly associated with the OS of advanced-stage SOC patients administered both platinum and taxol. The higher expression level of all 5 genes could predict shorter OS (Figure S3, Table 1). The higher expression level of 7 PFS-associated genes, BIRC5, CCNB2, CDC20, MYBL2, PLK1, TOP2A, and TTK, could predict longer PFS (Figure 8, Table 2).

### The differential expression of key genes between platinum-resistant and sensitive SOC samples

**Figure 9.**
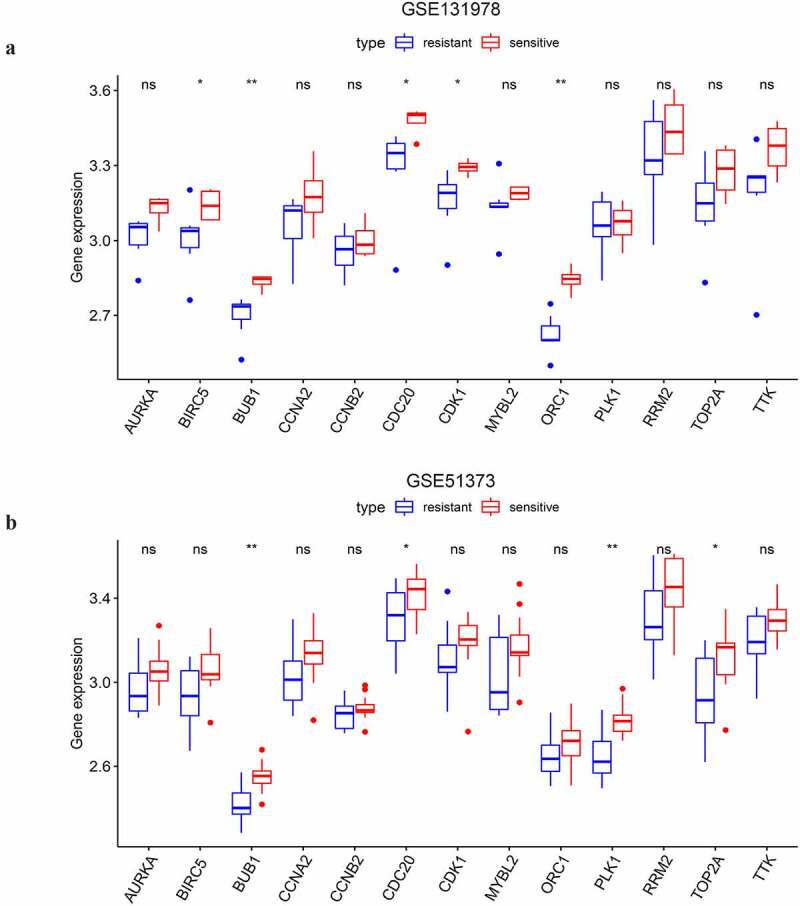
The differential expressional validation of the selected key genes between platinum resistant and platinum sensitive samples of advanced-stage SOC. (a) Differential expression of the key genes in the GSE131978 dataset. (b) Differential expression of the key genes in the GSE51373 dataset. ‘*’ indicates p < 0.05. ‘**’ indicates p < 0.01

As shown above, the 13 selected key genes were closely associated with platinum sensitivity. Thus, we selected two GEO datasets to illuminate whether the key genes played roles in the modulation of platinum response. The analysis on GSE131978 revealed that BIRC5, BUB1, CDC20, CDK1, and ORC1 had statistically significant differential expression between platinum-resistant and sensitive samples of stage III–IV SOC (Figure 9(a)). And the expression level of the 5 genes was upregulated in platinum-sensitive samples compared to platinum-resistant samples. In the analysis on GSE51373, we found that BUB1, CDC20, PLK1, and TOP2A had differential expression between chemotherapy (contains platinum) resistant and sensitive samples of advanced-stage SOC (Figure 9(b)). Consistent with the results of GSE131978, the 4 genes were also downregulated in chemotherapy-resistant samples compared to the chemotherapy-sensitive ones. BUB1 and CDC20 were the two overlapped DEGs of the two datasets. With PFS prognostic value, CDC20 was eventually considered a feasible indicator of platinum-based chemotherapeutic response.

## Discussion

SOC is a main histological subtype of OC with a poor prognosis. Debulking surgery combined with platinum-based chemotherapy is the primary therapy of SOC [25]. Although platinum-based therapy continued to be the first-line option of advanced-stage SOC, platinum is not the best approach for partial patients with limited platinum sensitivity (a platinum-treatment free interval of 6–12 months) [26]. Nowadays, poly (ADP-ribose) polymerase (PARP) inhibitors have provided great therapeutic benefits to OC patients. Platinum sensitivity is also a prospective biomarker for predicting the response to PARP inhibitors (PARPi) thus instrucing drug introduction of OC patients [27]. CSCs are a subpopulation of cancer cells closely correlated to survival time and therapeutic resistance of OC patients [28,29]. As the stemness of CSCs is an important cause of chemoresistance [30,31], investigating reliable drug response indicators, especially the indicators of platinum response among stemness-associated genes is feasible and essential. However, such platinum response indicators are still poorly understood. Although several studies have reported the use of biological information from different levels to obtain drug response indicators for OC [32]. Esra Gov recognized novel prognostic biomarkers in ovarian cancer by biological information [33]. In the study, we identified platinum-based chemotheraputic response indicator among stemness-associated key genes using a multi-step bioinformatics approach based on transcriptome sequencing.

Malta et al. had reported that a higher value of stemness indices was associated with greater tumor dedifferentiation, which was reflected by a higher histopathological grade [7]. Our results on the correlation between mRNAsi/corrected mRNAs and histopathological grades were very consistent with the concept proposed by Malta et al. In the PFS curves of mRNAsi/corrected mRNAsi, we observed a tendency without statistical significance that within approximately 4 years. This was different from our understanding that greater stemness of cancer cells would eventually lead to a shorter PFS [34]. The results might be explained by three possible causes. First, the absence of statistical significance might be caused by quite a small sample size included in the analysis. Second, the mRNAsi score was computed according to the transcriptomic characteristics of pluripotent stem cells and their differentiated progeny. Perhaps only a small part of genes involved in mRNAsi have a significant effect on the PFS of advanced-stage SOC patients. Thus, the analysis of the correlation between mRNAsi score and survival time could be influenced by a lot of confounding factors. Third, the overexpression of some stemness-associated genes involved in mRNAsi led to longer PFS of advanced-stage SOC patients. The third potential cause needed to be validated in the subsequent analyses.

WGCNA is a tool to analyze the gene expression pattern in multiple samples. It can classify those genes with similar expression patterns into clusters and further analyze the correlations between different gene clusters and certain characteristics [11]. In the WGCNA procedure, we chose the green module most positively correlated to mRNAsi to investigate stemness-associated biomarkers which possibly governed the stemness of SOC. The functional annotation of the selected key genes in the green module revealed that the genes were most enriched to the biological process of mitotic nuclear division, which was consistent with the top GO category of DEGs between OC and normal samples. This confirmed the uncontrolled cell proliferation was a core characteristic of OC cells as well as the ovarian cancer stem cells (OVSCSs) [35]. Previous studies reported that the pathway ‘Cell cycle’ [23,24], ‘Cellular senescence’ [19,20], ‘p53 signaling pathway’ [21,22], and ‘Platinum drug resistance’ were closely associated with platinum response. A recent review had summarized that an insufficient dose of platinum might lead to a cytostatic response, named dormancy, rather than cytotoxic response, through inducing cell cycle arrest and cellular senescence [36]. Therefore, investigating platinum-response indicators among the key genes enriched to the platinum-response associated pathways could be reliable.

AURKA, MYBL2, ORC1, and PLK1 were significantly correlated with the OS of advanced-stage SOC patients in our results. AURKA targeting inhibits self-renewal capacity and restores sensitivity to DTX-based chemotherapy in breast cancer [37]. PLK1 was also reported to promote Epithelial-Mesenchymal Transition (EMT), a biological process that was closely associated with cell stemness, in gastric carcinoma cells [38]. These reports confirmed that AURKA and PLK1 were potential modulators of the stemness of SOC. However, MYBL2 and ORC1 hadn’t been reported whether associated with cancer stemness. Among the 11 key genes associated with platinum response investigated by the PFS analyses, higher expression of all these genes but ORC1 could predict longer PFS of advanced-stage SOC. It was very interesting that higher expression of AURKA, TOP2A, and TTK could predict longer PFS but shorter OS of patients receiving platinum and taxol. This indicated that these genes could promote tumor progression as oncogenes while prolonging PFS as a drug sensitivity biomarker. However, the underlying mechanism of how the genes impacted OS and PFS in a reverse pattern still needed to be further illuminated. Among the PFS-associated genes, AURKA [22,39], CDK1 [40], and RRM2 [12] had been validated correlating to the platinum response by experimental methods. These reports partially verified that identifying platinum-response indicators among stemness-associated genes according to mRNAsi was reliable and worthwhile. However, the function of the genes on maintaining stemness and modulating the platinum response of ovarian cancer cells still needs to be verified by experimental methods.

Results of this study revealed that BUB1 and CDC20 had significantly higher expression levels in platinum-sensitive samples than that of platinum-resistant in both two GEO datasets. Accumulating evidence indicates that there is a strong link between abnormal upregulation of CDC20 and various types of tumors [41–43]. CDC20 knockdown was shown to sensitize cancer cells to chemotherapy and radiation therapy [44,45]. CDC20 overexpression facilitates the docetaxel resistance of the advanced castration-resistant prostate cancer cell lines [46]. Moreover, results from recent TCGA and pathological studies have demonstrated a pivotal oncogenic role for CDC20 in tumor progression as well as drug resistance [47]. BUB1 is an independent prognostic indicator for ovarian cancer and was found depleted in paclitaxel resistant human ovarian cancer cells [48,49]. BUB1 was not included in the results of PFS significance analysis in this study. With significant prognostic value, CDC20 was considered a potential response indicator to platinum-based chemotherapy. There are also some limitations of this research. First, compared with tumor tissue samples, the sample size of normal tissue in the GTEx database is small. Although multiple prognostic markers of OC have been identified based on the GTEx database [50–52], we need to further expand the sample size from our center in the future. Second, the generation of chemoresistance was a complex process involving networks of genes and pathways. A single gene would not be precise enough to predict drug response. Investigating drug response indicators by multiple methods, constructing a drug-response prediction model with multiple genes, and validating the effectiveness of the drug response indicators by experimental methods would be carried out in our next task.

## Conclusion

In conclusion, we investigated platinum-response indicators among stemness-associated key genes in advanced-stage SOC according to mRNAsi in this study. By evaluating the prognostic value and expressional validation, CDC20 was identified as a stemness biomarker and platinum-response indicator in advanced-stage SOC. This conclusion would provide clues to guide clinical drug use and still needs to be further validated by experimental methods.

## Supplementary Material

Supplemental Material

## Highlights

1. Serous ovarian cancer of higher histopathological grades had greater stemness indices
2. Forty-four genes positively correlated to stemness characteristics advanced-stage SOC
3. Thirteen genes involved in platinum response were identified in multiple databases
4. CDC20 was identified as a platinum-based chemotherapy indicator in SOC
