# Supplementary material for: Transcriptome-based stemness indices analysis reveals platinum-based chemo-theraputic response indicators in advanced-stage serous ovarian cancer
Source: Bioengineered. 2021 Jul 16;12(1):3753–71. doi: 10.1080/21655979.2021.1939514 (PMC8806806; doi:10.1080/21655979.2021.1939514)
Supplement: Supplemental Material [file KBIE_A_1939514_SM3164.zip › supplementary/downloadFromZipFile11.pdf]

Supplementary Figures

Figure S1

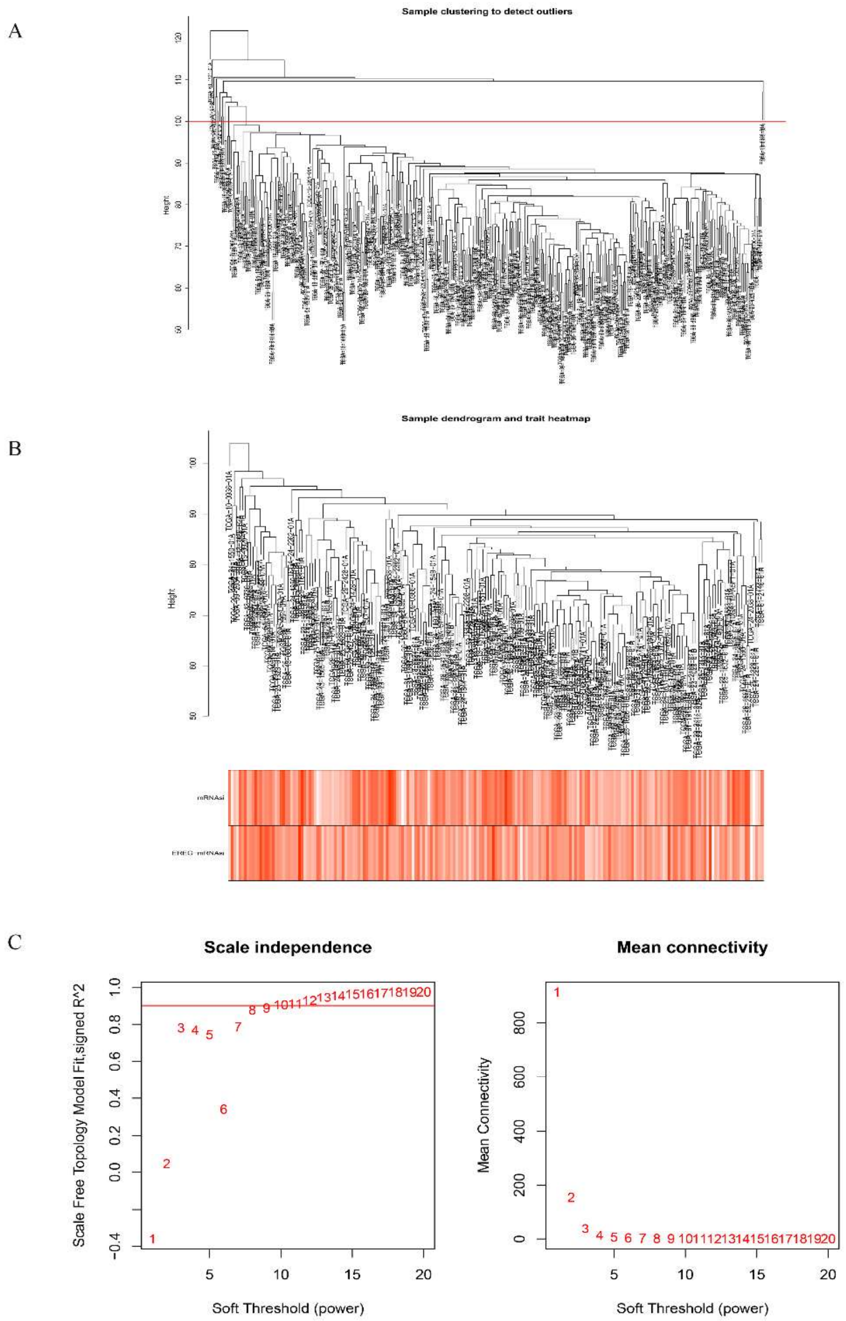

Figure S1: The preparation procedures of WGCNA. (A) Removal of 15 outlier samples. The cutheight was set at 100; (B) Cluster dendrogram of remained 337 SOC samples of stage III-IV according to mRNAsi and EREG-mRNAsi; (C) Determination of soft threshold and inspection of scale-free network.

Figure S2

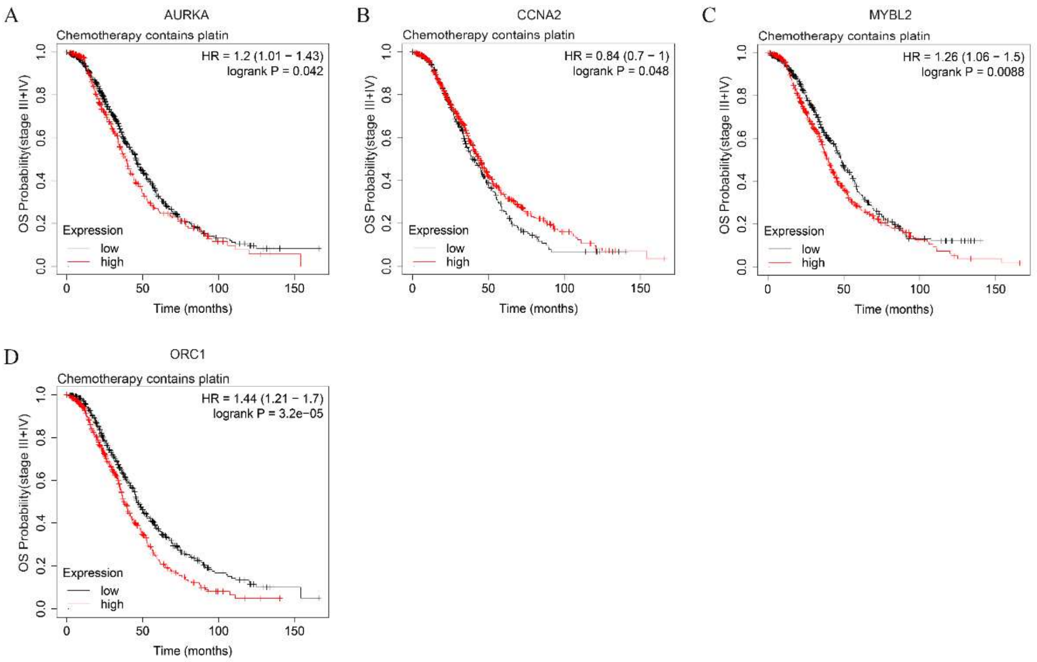

Figure S2: The OS curves of 4 key genes with significant prognostic value of overall survival time analyzed on 936 SOC patients of stage III-IV treated with chemotherapy containing platin. $p<0.05$ indicates statistical significance.

Figure S3

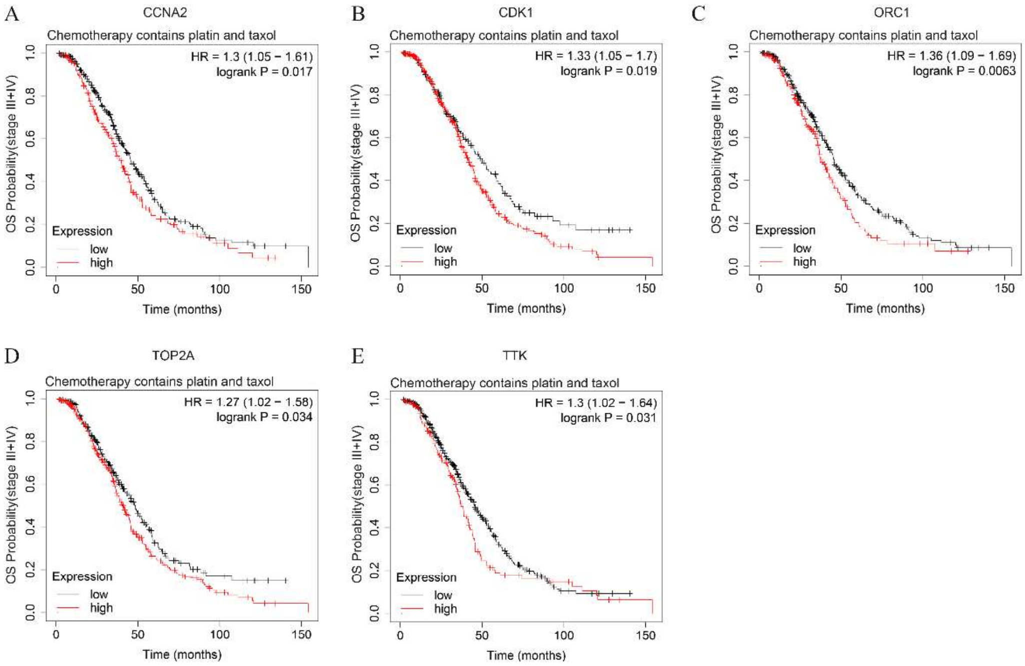

Figure S3: The OS curves of 5 key genes with significant prognostic value of overall survival time analyzed on 572 SOC patients of stage III-IV treated with chemotherapy containing both platin and taxol. $p<0.05$ indicates statistical significance.
